# Attentional Capture and Inhibition of Saccades after Irrelevant and Relevant Cues

**DOI:** 10.1155/2014/585921

**Published:** 2014-04-22

**Authors:** Heinz-Werner Priess, Nils Heise, Florian Fischmeister, Sabine Born, Herbert Bauer, Ulrich Ansorge

**Affiliations:** ^1^Faculty of Psychology, University of Vienna, Liebiggasse 5, 1010 Wien, Austria; ^2^MR Centre of Excellence, Medical University of Vienna, 1090 Wien, Austria; ^3^Study Group Clinical fMRI, Department of Neurology, Medical University of Vienna, 1090 Wien, Austria; ^4^Laboratoire Psychologie de la Perception, Université Paris Descartes, 75006 Paris, France; ^5^Institute of Cognitive Science, University of Osnabrück, 49069 Osnabrück, Germany

## Abstract

Attentional capture is usually stronger for task-relevant than irrelevant stimuli, whereas irrelevant stimuli can trigger equal or even stronger amounts of inhibition than relevant stimuli. Capture and inhibition, however, are typically assessed in separate trials, leaving it open whether or not inhibition of irrelevant stimuli is a consequence of preceding attentional capture by the same stimuli or whether inhibition is the only response to these stimuli. Here, we tested the relationship between capture and inhibition in a setup allowing for estimates of the capture and inhibition based on the very same trials. We recorded saccadic inhibition after relevant and irrelevant stimuli. At the same time, we recorded the N2pc, an event-related potential, reflecting initial capture of attention. We found attentional capture not only for, relevant but importantly also for irrelevant stimuli, although the N2pc was stronger for relevant than irrelevant stimuli. In addition, inhibition of saccades was the same for relevant and irrelevant stimuli. We conclude with a discussion of the mechanisms that are responsible for these effects.

## 1. Introduction

Visual attention is the selection of visual information for purposes such as in-depth processing, perception, or action control. Because we have to select information at all times, understanding attention is a key to an understanding of almost any form of cognition. To date, however, the mechanisms by which attention operates are not fully understood.

One persistent debate in this area concerns the role of inhibition of irrelevant stimuli as one form of top-down control over attention. Whereas some researchers believe that inhibition of attention is a response to initial capture of attention and, thus, follows preceding attentional capture by an irrelevant stimulus [1], other researchers believe that active inhibition of attentional capture by an irrelevant stimulus is possible right from the start of such a stimulus [2].

To start with the first proposition, many researchers argued that salient objects capture attention in a bottom-up way (cf. [3, 4]). According to the salience model of attention, any visual stimulus that stands out among its surroundings by a strong feature contrast in color, orientation, or luminance may capture attention in an exogenous stimulus-driven way, regardless of the current goal of the observer (cf. [5, 6]). In line with this prediction, an irrelevant color singleton distractor—that is a stimulus with a color different from its surrounding stimuli, such as one green circle among several red circles, interferes with finding a shape-defined target stimulus (i.e., the one rectangle among several circles) (cf. [4]). This is the case although attending to the specific color of the singleton is neither necessary nor helpful for finding the target. Such findings have been attributed to the bottom-up capture of attention by an irrelevant singleton. As a consequence, attention is thought to first be distracted away from the relevant target and only later be redirected towards the target. This is possible after a deliberate inhibition of the irrelevant stimulus, allowing attention to disengage from the distractor [1].

Findings by Kim and Cave [7] are in general agreement with this late-inhibition or disengagement hypothesis. These authors used a probe stimulus as a second target in a combined search and probe reaction task. Critically, the probe was shown after the search display with the probe either at the position of the search display's shape-defined target or at the position of the search display's color-singleton distractor. With an interval of 60 ms between search display and probe, Kim and Cave observed (nonsignificantly) faster responses to probes at color-distractor positions than to probes at shape-target positions. However, with a cue-target onset asynchrony (CTOA) of 150 ms between the color-singleton distractor (cue) and the probe (target), responses to probes at the position of the color-singleton distractor were significantly* delayed* relative to responses to probes presented at the location of the shape target. This delay evidently reflected active inhibition of the color distractor that developed over time. Like Theeuwes et al. [1], Kim and Cave [7] took their results as an indication of bottom-up capture by the color-singleton distractor with a short CTOA, giving way to disengagement and even active inhibition with a long CTOA. The core notion of the disengagement hypothesis, that is, the idea of active inhibition following initial allocation of attention towards a stimulus, is also at the heart of another well-known phenomenon called inhibition of return (IOR). IOR denotes the finding that attracting visual attention toward one position in space by a cue delays a second attention shift to the same position at a later point in time [8–10]. IOR is observed with long CTOAs and corresponds to longer reaction times where cue and target are presented at the same position (SP) compared to cue and target at different positions (DP). Thus, the idea of stimuli initially triggering attentional capture and later inhibition is a very dominant notion found throughout the attention literature.

**Figure 1:**
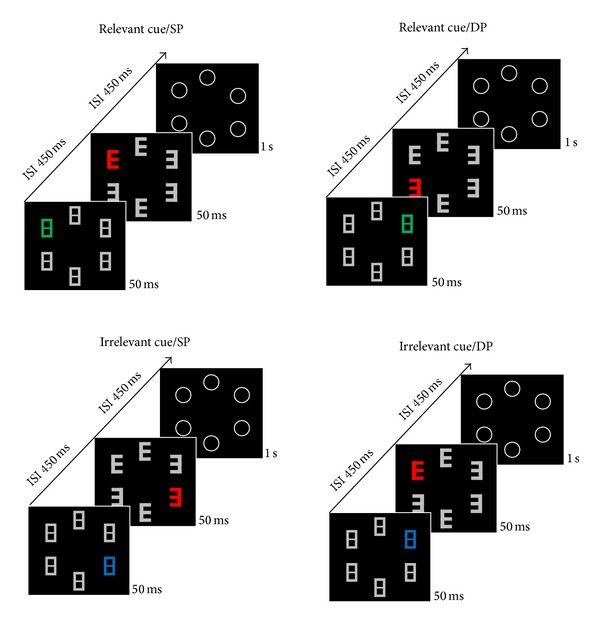
Depicted are examples of a different-position (DP) trial with a relevant cue (in the upper right), a same-position (SP) trial with a relevant cue (in the upper left), a different-position (DP) trial with an irrelevant cue (in the lower right), and a same-position (SP) trial with an irrelevant cue (in the lower left). In each of the four depicted conditions, the first (lower) display was the cue display, in which a color singleton cue (illustrated as a green or blue shape-8) was presented. The second (middle) display was the discrimination display where participants had to memorize the identity (i.e., shape-*E* or shape-3) at the position of a relevant cue (here: green) but not after an irrelevant cue (here: blue) in the first display. Alongside the discrimination target, we presented a red color singleton as a saccade cue. The third (upper) display was the saccade display; participants had to saccade to the target ring at the position cued by the red singleton. The arrow illustrates the temporal sequence. Stimuli are not drawn to scale. ISI = interstimulus interval.

However, recent findings by McDonald et al. [2] and by Ansorge et al. [11] are potentially in disagreement with this late-inhibition or disengagement hypothesis for irrelevant stimuli. Ansorge et al. asked their participants to saccade to one out of four positions, varying randomly from trial to trial (see Figure 1 for an illustration of a similar stimulus and task sequence). Prior to the saccades, participants were presented with a relevant or an irrelevant color singleton cue. Participants only had to attend to the relevant cue because this cue indicated the position of a discrimination target later in the trial. In contrast, the participants were asked to ignore the irrelevant cue: when an irrelevant cue was presented, no target discrimination was required so that it was safe to ignore this cue. In addition, ignorance of the irrelevant cue was encouraged: because cue and saccade target positions were uncorrelated (cf. [12]) and because saccades require a prior attention shift to the target position [13, 14], the participants could fully concentrate on the saccade task and ignore the irrelevant cues completely. In contrast, the participants were forced to shift their attention to the relevant cue for the encoding of its position for the discrimination task and at the potential cost of a suboptimal preparation of their saccades. All of these cues were nonpredictive of the saccade target position, and relevant and irrelevant cues had different fixed colors, so that the participants knew exactly which color they had to attend to (e.g., red) and which color they could ignore (e.g., green). Under these conditions, Ansorge et al. [11] studied the time course of selective attentional capture and/or inhibition by looking at the development of the saccadic latencies across the latency distribution, from quick to slow saccades. With a long CTOA, and with relevant cues, IOR followed initial capture: initial capture among the fast responses was reflected in quicker saccades to a saccade target at the same position (SP) as the cue compared to slower saccades to a saccade target at a different position (DP) than the cue. With relevant cues, this pattern reversed into IOR among the slower responses. In contrast, with a long CTOA and irrelevant cues, inhibition in the form of slower saccades to SP than DP targets was found right from the beginning and without preceding capture effects. These findings point to a form of proactive inhibition of irrelevant cues, completely preventing attentional capture by the irrelevant cues, rather than late disengagement. In fact, the only reliable capture effect for irrelevant cues showed up in a condition with a different procedure and no subsequent inhibition (experiment 4). Thus, no conclusion could be drawn about transitions from capture to disengagement.

A recent study by McDonald et al. [2] equally found evidence for proactive inhibition of irrelevant stimuli without a trace of preceding attention capture. To discern between capture and inhibition, McDonald and colleagues used two lateralized components of the event-related potential (ERP): the N2pc (cf. [15, 16]) or posterior contralateral negativity (PCN) [17] and the Pd [18, 19]. The N2pc has been widely used to investigate both stimulus-driven capture [20] and top-down contingent capture [21–25]. It is a larger negative deflection contra- than ipsilateral to an attended stimulus. It occurs approximately 200–280 ms after stimulus onset over posterior areas [26]. In contrast, the Pd reflects the active inhibition of potentially distracting stimuli [19]. It is a component of similar latency and scalp distribution as the N2pc. However, it is of opposite polarity as compared to the N2pc. Importantly, when McDonald et al. [2] tested for initial capture of attention by irrelevant stimuli, all they found was a Pd, that is, evidence for proactive inhibition of the irrelevant stimuli. This was found after splitting the ERPs into fast and slow responses: the quickest target responses of the participants indicated proactive inhibition of attentional capture by an irrelevant distractor.

Even so, it is not entirely clear whether the findings reflected only early inhibition or whether some capture of the irrelevant singletons occurred before it was suppressed. Regarding the findings of Ansorge et al. [11], these authors used saccadic latencies after relatively long CTOAs (>200 ms). This method is relatively insensitive to the early attentional effects, so that preceding attention capture even by irrelevant cues might have gone unnoticed (see their condition with a CTOA of 200 ms). Regarding the findings of McDonald et al. [2], it is possible that their observations reflected a mixture of weaker capture effects of the irrelevant distractors in some of the trials and of stronger inhibition of distraction in other trials. As a result, a net inhibitory Pd effect could have masked evidence for early capture in the form of an N2pc in the study of McDonald et al. At least in the slower responses, there was also clear evidence for this possibility: “On slow response trials (…) there was neither an early distractor (…) nor a late target N2pc (…). The absence of either N2pc suggests that the target and distractor N2pc wave cancelled each other out (…)” (p. 856, McDonald et al.). Thus, to test once more whether capture by irrelevant cues could precede subsequent inhibition, we combined the methods of Ansorge et al. and of McDonald et al., using two different measures for early and late effects based on the very same trials. We used ERPs to assess early effects of capture or proactive inhibition. Late inhibition was assessed through the presence of IOR in saccadic reaction times after a sufficiently long CTOA of 1 s. This procedure allows for the registration of an early capture effect by all singletons, without masking by a concomitant N2pc by the targets [27]. In this situation, initial capture by the irrelevant singleton cue should show up as an N2pc. In addition, we conducted a median-split of the ERPs on the basis of whether a fast or a slow saccade was given that allowed us to test whether the fastest responses were associated with a Pd component, similar to McDonald et al. [2].

## 2. Experiment

The aim of the current experiment was to investigate the connection between attention capture by relevant and irrelevant stimuli and (subsequent) inhibition. We examined within the same trials (1) the amount of initial capture of attention by an irrelevant cue and a relevant cue—in the form of the N2pc—and (2) the amount of inhibition—in the form of an early Pd and late saccadic inhibition of return.

In detail, in the first display of each trial, we used one of two color-singleton cues: the first cue was relevant in half of the trials and it was irrelevant in the other half of the trials. A relevant first cue had a fixed color (e.g., it was green), known to the participant. The participant had to look for the relevant first cue and importantly, also* covertly,* attend to its* location* because it indicated the position of a subsequent discrimination target. We call this cue “relevant” to make clear that its color serves the same purpose as a searched-for feature of a target in a standard color-search task. The relevant cue indicated with 100% certainty the position of the discrimination target.

In contrast, the irrelevant first cue had a different color (e.g., it was blue if the relevant cue was green), also known to the participants. No target discrimination was required after an irrelevant first cue. Therefore, the participants could have ignored this cue completely and were not required to shift spatial attention to its location. We call this cue “irrelevant” because its color served the same purpose as the color of an irrelevant distractor in a visual search display. That is, the color of the irrelevant first cue indicated with 100% certainty that this stimulus could be safely ignored.

To test IOR we introduced a later secondary saccade task. After the first cue, our participants had to encode the position of a second singleton cue in a second display for a saccade in a subsequent third display (see Figure 1). This second or saccade cue was always red. Importantly, positions of the first cue and of the second saccade cue were uncorrelated. Because the participants have to allocate their attention to the position of the saccade target (cf. [13, 14, 28, 29]), they had to disengage their attention away from any first cue and to redirect it towards the position of the second cue in anticipation of the saccade target. Also, the CTOA was 1 s long allowing for both inhibition (or disengagement) of attention and saccadic inhibition (of return). We therefore expected saccadic inhibition with respect to the position of the first or covert cue ([11, 30, 31]; see also [32]). The question is whether with irrelevant first cues a capture effect in the form of an N2pc precedes this inhibition effect or whether inhibition is observed from the start, in the form of a Pd. Also, in the relevant condition, an N2pc to the first cue was to be expected because an attention shift to this first cue was required to encode its position.

### 2.1. Materials and Method

#### 2.1.1. Participants

Twelve volunteers participated but one was excluded because her saccade latencies were more than three standard deviations slower than that of the other participants. The remaining participants (with a mean age of 25 years and a male/female ratio of 6 : 5) reported normal or corrected-to-normal vision. Written and informed consent was obtained from each participant before the experiment.

#### 2.1.2. Stimuli and Procedure

Visual stimuli were presented on a 19-inch CRT color monitor (Sony Multiscan G400), with a screen resolution of 1,024 × 768 pixels and a refresh rate of 100 Hz. The participants sat at a distance of 57 cm from the screen in a quiet, dimly lit room, with their head resting on a chin rest to ensure a constant viewing distance and a straight-ahead gaze direction.

Three successive displays were shown on each trial (see Figure 1). The first and second displays were presented for 50 ms and the last display for 1 s. All displays were separated by an interstimulus interval of 450 ms, such that the onset asynchrony between two displays was 500 ms. A gray central fixation cross was presented on a black background (<1 cd/m²), visible throughout each trial. All objects on the screen were equiluminant (~30 cd/m²).

The first display consisted of six equidistant placeholders, each in the shape of the digital letter 8 (with a size of 1.7° × 1° and with stroke strength of .3°). A placeholder was located per each of the positions at 0°, 60°, 120°, 180°, 240°, and 300° from the vertical meridian—that is, the shape-8s were presented equally spaced on the circumference of a virtual circle centered on the screen, with an eccentricity of 7°. Five placeholders were presented in gray (CIELAB color coordinates: 6.9, 16.8), and one was presented in a different color, either in green (CIELAB: −30.2, 24.9) or blue (CIELAB: 46.9, −89). This green or blue stimulus was the first color-singleton cue. It was always shown at one of the four lateral positions but never presented above or below the fixation.

Following the first display, the discrimination display was presented for 50 ms. The shape-8s were replaced by three letters “*E*” and three digits “*3*”, in digital notation. Five of these shapes were presented in gray and one was presented in red (CIELAB: 47.6, 41.1). This red stimulus was the second or saccade cue and it could also only appear at one of the four lateral positions. The red singleton was called a saccade cue because this second cue served as the cue for the saccade target in the subsequent display. Also, in this display, one figure served as a discrimination target if it had been cued by a relevant first cue (blue or green cue) in the preceding display, with relevant first cue color fixed across trials and balanced across participants. Positions of the discrimination target and second (or red) cue were uncorrelated across trials. Consequently, in 25% of the trials the discrimination target and second or saccade cue were at the same position (SP condition), and in 75% of the trials they were at different positions (DP condition).

Following another interval of 450 ms the saccade display was presented. This display consisted solely of six empty circles surrounding the stimulus positions as used in the preceding displays. The saccade display was presented for 1 s.

The color of the first singleton cue in the first screen indicated whether the discrimination task in the second screen had to be performed on a given trial. For instance, a first green singleton was linked to the discrimination task while a first blue singleton could be ignored, or vice versa. In the discrimination task, participants had to encode and remember the shape of the digit presented in the second screen at the position of the relevant first singleton cue. This was necessary for the report of this figure at the end of the trials. The second or red singleton cue indicated the position of the subsequent saccade target. As soon as the third display, the saccade display, appeared the saccade had to be executed. After the saccade was executed, in a relevant-cue trial, participants typed the identity of the discrimination target letter (i.e., whether the letter *E* or the digit* 3* was presented) by pressing the marked buttons #*F* and #*J* labeled “left” and “right” on a standard keyboard directly in front of the participants. If no discrimination was necessary (i.e., after irrelevant cues), this part of the trial was skipped. Participants started the next trial in a self-pace manner, by pressing the space bar. After this, 500 ms elapsed before the presentation of the cue display.

Participants were informed that the color singleton cues could only appear at the four lateral positions on the screen and that the position of the second or saccade singleton cue was independent of the position of the first singleton cue. Blocks consisted of 64 trials and feedback was given about whether the target discrimination was correct and about whether the saccade was registered during the third screen. Altogether ten blocks of trials were conducted, of which the first was training and not analyzed. Each factor combination of the variables discrimination target (*E* or* 3*), first cue position (above/left, above/right, below/left, and below/right), first cue color (blue, green), and second cue's position (above/left, above/right, below/left, and below/right) was equally likely and presented in a pseudorandom order within each block.

#### 2.1.3. Eye-Tracking and Saccade Analysis

Saccades were recorded with an EyeLink 1000 Desktop Mount system (SR Research, Mississauga, ON, Canada) with a 35 mm lens and EyeLink Software version 4.52, sampling at 1,000 Hz. Eye-tracking was monocular from the dominant eye. A 9-point calibration was used to adjust the eye-tracker before the experiment and in advance of every single block. Saccadic reaction time (saccadic RT) was calculated as the time between (1) the onset of the third display (with the saccade-target stimulus circle) and (2) the time of a local velocity minimum that immediately preceded the point in time at which eye velocity exceeded 80°/s. Only trials with correct saccades were analyzed. A saccade counted as correct if it landed in an area of 1.5° around the center of the saccade target. Saccade landing position was calculated as the *x*-*y* coordinates of the eye-tracker signal at the time at which eye velocity returned to a presaccadic baseline level. Also, if the eyes started to move earlier than 100 ms after the saccade target, a trial was discarded.

#### 2.1.4. EEG Recording and Analysis

DC-EEG was recorded from 23 scalp electrodes mounted in an elastic cap at standard positions of the extended 10/20 system at sites Fpz, F7, F3, Fz, F4, F8, Fc5, Fc6, T7, C3, Cz, C4, T8, Cp5, Cp6, P7, P3, Pz, P4, P8, O1, O2, and Oz. The continuous EEG was sampled at a rate of 1,000 Hz with a digital low-pass filter of 50 Hz. Impedance was kept below 2 kΩ. No further filters were applied after EEG acquisition. All scalp electrodes were online referenced to a noncephalic sternovertebral site, above the seventh vertebra and the right manilum sternum [33]. The vertical EOG (electrodes below and above the left eye) and the horizontal EOG (electrodes at the outer canthi) were recorded bipolarly, so as to delete trials with eye movements during the critical EEG recording interval. Trials with saccades earlier than 100 ms after the saccade target (detected with the eye-tracker) or muscular artifacts (exceeding ±80 *μ*V at any electrode), as well as trials in which the target was not correctly discriminated, were excluded from analysis. ERPs were calculated for 400 ms after the first cue's onset relative to a 50 msec precue baseline. N2pc amplitudes in response to the first color cue were calculated separately for left and right and relevant and irrelevant cue, collapsed across all saccade target positions as mean ERP amplitudes at locations P3/4 in the 160–270 ms interval after cue onset.

#### 2.1.5. Synchronization of Eye-Tracking and EEG

A switch box was implemented behind the parallel port of the master to send one unique synchronization trigger every 500 ms (one for the onset of the first display, one for the second display, and one for the third display in each trial) in parallel, separately to the two slaves, eye-tracker and EEG recorder.

### 2.2. Results

In total, 17.5% of all trials were excluded. Trials with saccades faster than 100 ms and slower than 1 s after the saccade target accounted for 8.1%, trials with saccades towards the wrong target or with muscular artifacts for another 6.4%, and trials with a false identification of the discrimination target for 3%.

#### 2.2.1. Saccade Task

To take the dynamics of the saccadic response into account, saccadic RTs were sorted and grouped into five percentiles from fast to slow (cf. [31]). This was done to test our hypotheses about IOR with differently fast responses because the amount of capture and of IOR does vary over time and an effect that is absent in the average of all responses can well be present when looking at only the faster or only the slower responses (e.g., [11, 34]).

**Figure 2:**
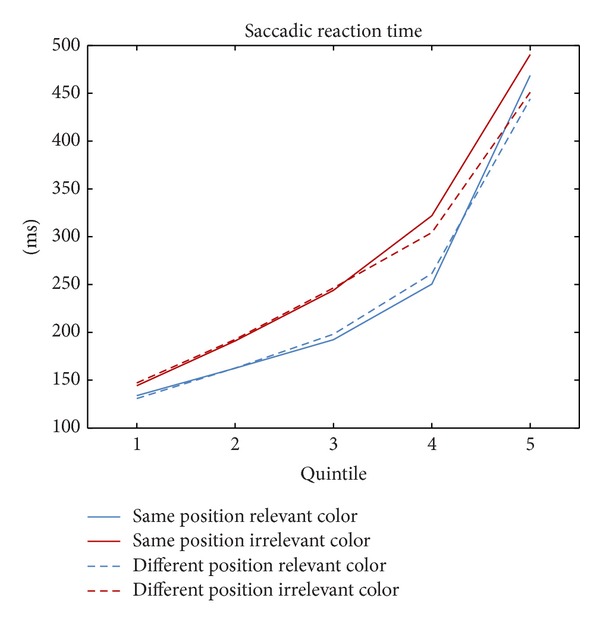
Saccadic reaction time (SRT) in milliseconds as a function of the first cue's relevance (relevant cue = blue lines, irrelevant cue = red lines), cue position (at the same position as the target = solid lines, at a different position than the target = dashed lines), and the quintile of the SRT distribution (1 to 5, from fastest to slowest).

As can be seen in Figure 2, from fast responses on the left to slow responses on the right, there was a gradual build-up of IOR. This was reflected in faster saccadic RTs under DP conditions (broken lines) as compared to SP conditions (solid lines), more so with the irrelevant cues (red lines) than with the relevant cues (blue lines).

A repeated-measures ANOVA with the variables position (same versus different position of first or covert cue and saccade cue/target), cue type (relevant first cue or irrelevant first cue), and percentile (1st to 5th) revealed inhibition at the location of the first or covert cue only among the slowest responses in the form of slower saccadic latencies in SP than DP conditions. This was reflected in a significant interaction of position and percentile, *F*(4,40) = 2.79, *p* < .05. From the 1st to the 5th quintile, saccadic inhibition (saccadic RT in SP conditions minus saccadic RT in DP conditions) was 0 ms, −1 ms, −4 ms, 3 ms, and 32 ms (1st to 4th quintile, all *t*s < 1; 5th quintile, *t*(9) = 2.29, *p* < .05). In addition, we found faster saccadic RTs in trials with a relevant than an irrelevant cue in the first display (241 ms versus 273 ms), resulting in a marginally significant main effect for cue type, (1,10) = 4.72, *p* = .055. There was also a trivial main effect of percentile (increasing saccadic RTs with percentile), (4,10) = 94.56, *p* < .01.

Further, there was a numerically stronger inhibitory effect on saccades after the irrelevant cue (10 ms) than after the relevant cue (2 ms), as would be expected based on an active inhibition explanation. However, the two-way interaction of relevance and position was not significant, (1,10) = .55, *p* = .48, as was the three-way interaction, *F* < 1. In sum, saccadic inhibition was selectively present in the slowest saccades and it was largely independent of the type of cue that was used in the first display.

#### 2.2.2. N2pc to the First Cue

**Figure 3:**
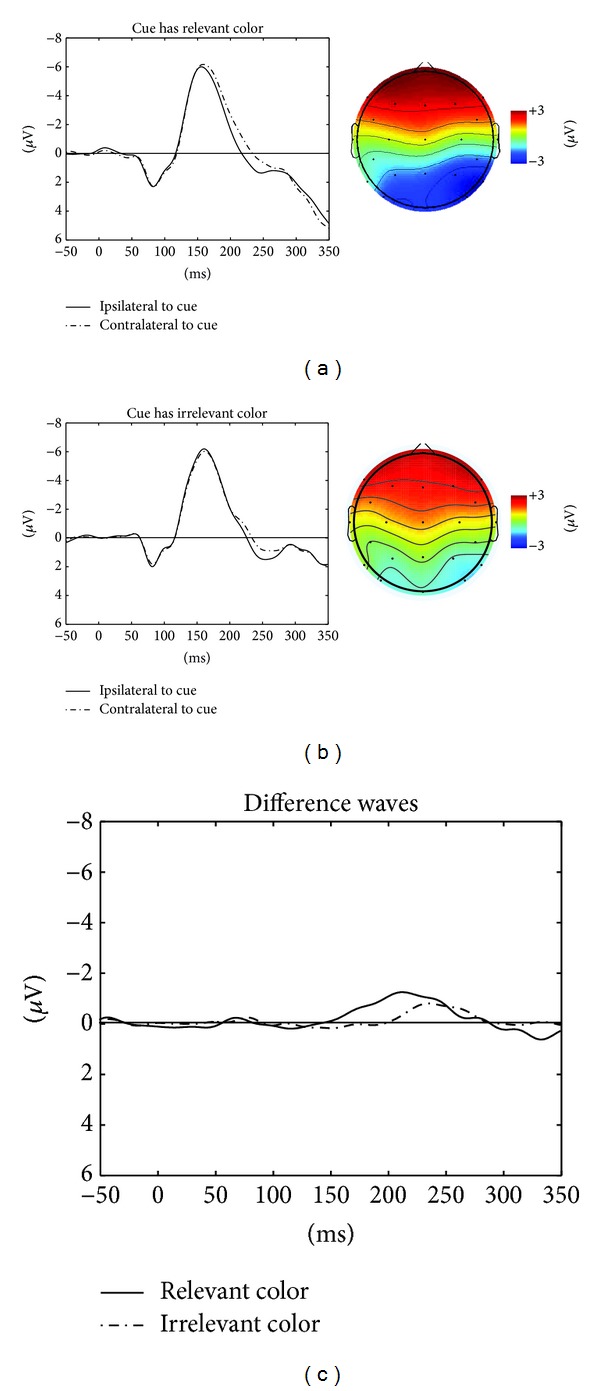
(a) ERPs (in *μ*Volts on the *y*-axis) ipsilateral to the relevant cue (solid line), contralateral to the relevant cue (dashed line) as a function of the time since cue onset (at zero) on the *x*-axis, and scalp distribution plots of mean ERP activity in response to the cues in a time window of 160 to 270 ms after the cue, with contralateral activity to the right and ipsilateral activity to the left and with negative values in blue and positive values in red. (b) The same as (a) for irrelevant cues. (c) Difference waves of contralateral minus ipsilateral activity, separately for relevant cues (solid line) and for irrelevant cues (dashed line).

Figure 3 shows ERPs time-locked to the first cue's onset at lateral posterior electrodes P3 and P4 contra- and ipsilateral to the first cue, separately for cues with a relevant color (panel a), cues with an irrelevant color (panel b), and difference waves (i.e., contra- minus ipsilateral activity for relevant and irrelevant cues, panel c). The differences are depicted together with topographical ERP-difference maps for the time window of the N2pc (160 ms to 270 ms). All ERPs are relative to a baseline from −50 ms before the first cue to the onset of the first cue. As can be seen, there was an N2pc in the relevant and in the irrelevant cueing conditions. Also, by looking at Figure 3, it seems as if the N2pc started later and was weaker in the irrelevant than in the relevant cueing condition.

These observations were confirmed in a repeated-measures ANOVA with the variables cue type (relevant or irrelevant cue), laterality (electrode ipsi- or contralateral to the first cue), and hemisphere (right or left hemisphere). The analysis revealed a significant main effect for laterality, (1,10) = 7.3, *p* < .05, and a significant interaction of laterality and cue type, *F*(1,10) = 10.14, *p* < .01. Cues elicited an N2pc, regardless of whether the cue was relevant or irrelevant. However, if the cue was relevant, the N2pc was stronger (contra- minus ipsilateral activity: 0.76 *μ*V) and started earlier than if the cue was irrelevant (.30 *μ*V), as was shown in Section 2.2.3.

We were concerned that the choice of the electrode locations of the N2pc might have been unfortunate. Therefore, we repeated our major analysis of the N2pc in a repeated-measures ANOVA with the additional variable site (P3/P4, P7/P8, and O1/O2) and the variables cue type (relevant or irrelevant cue) and laterality (electrode ipsi- or contralateral to the first cue) as before. Besides replicating the main effect of laterality, *F*(1,10) = 6.86, *p* < .05, and an interaction of laterality and cue type, *F*(1,10) = 5.56, *p* < .01, there were no significant main effects, all *F*s < 1.40 and all *p*s > .28, and no significant interactions including the three-way interaction of site, cue type, and laterality, all *F*s < 2.10 all *p*s > .15. In addition, the ANOVA was also repeated with the ERPs pooled across P3, P7, and O1 (for the left side) and across P4, P8, and O2 (for the right side). This ANOVA also confirmed a laterality effect, (1,10) = 6.86, *p* < .05, and an interaction of laterality and cue type, *F*(1,10) = 5.56, *p* < .01, and no main effect of cue type, *F* < 1.

#### 2.2.3. N2pc to the First Cue: Early Phase

To demonstrate the earlier onset of the N2pc with relevant cues than with irrelevant cues, we split the N2pc window into an early phase (160 ms to 215 ms after the cue onset) and into a late phase (215 ms to 270 ms after the cue onset; cf. [35]). In the early window, an ANOVA revealed a significant two-way interaction of laterality and cue type, (1,10) = 30.17, *p* < .01. Post-hoc *t*-tests revealed that the contra-to-ipsilateral negativity difference (−.78 *μ*V) was only significant in the relevant condition, (10) = 4.33,* p *< .01, but not in the irrelevant condition (.02 *μ*V), *t*(10) = .08,* p *= .93.

#### 2.2.4. N2pc to the First Cue: Late Phase

A similar ANOVA of the late time window only led to a main effect of laterality, (1, 10) = 8.10, *p* < .05. The contra-to-ipsilateral negativity difference was about similar in relevant (.77 *μ*V) and irrelevant (.66 *μ*V) cueing conditions. There was neither a main effect of cue type, nor of hemisphere, nor any interaction between the variables, all other *F*s < 2.10 all *p*s > .18.

#### 2.2.5. N2pc to the First Cue: Fast Responses

**Figure 4:**
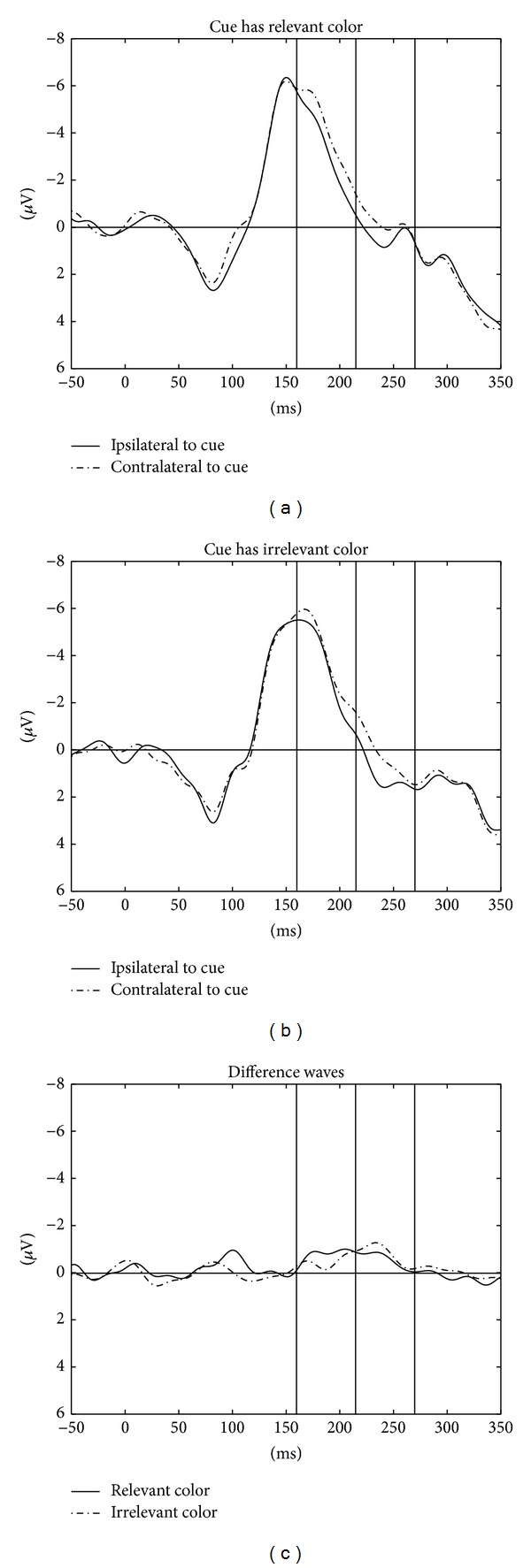
Data from the 50% fastest responses. (a) ERPs (in *μ*Volts on the *y*-axis) ipsilateral to the relevant cue (solid lines) and contralateral to the relevant cue (dashed lines), as a function of the time since cue onset (at zero) on the *x*-axis. (b) Same as (a) but for irrelevant cues. (c) Difference waves of contralateral minus ipsilateral activity, separately for relevant cues (solid line) and for irrelevant cues (dashed line).

Recently, McDonald and colleagues [2] showed that irrelevant distractors elicited a Pd among the fastest responses. We therefore also repeated our ANOVA of the activity at P3 and P4, with only the fastest 50% of the saccades and the two within-participant variables cue type (relevant or irrelevant cue), and laterality (electrode ipsi- or contralateral to the first cue). Again, activity was more negative at contra- than ipsilateral electrodes, (1, 10) = 11.16, *p* < .05. This time, however, the interaction was far from significant, *F* < 1. In contrast to the findings of McDonald et al. [2], a more prominent N2pc rather than a Pd was observed with the irrelevant singleton cues during the fastest responses. This can also be seen by looking at Figure 4.

## 3. Discussion

In the present study, we tested whether irrelevant cues were proactively inhibited or whether they captured attention before being inhibited. In line with the latter possibility, relevant, and importantly also irrelevant, cues elicited an N2pc and both stimuli led to inhibition of saccades 1 s after the cues. This was reflected in slower saccadic RTs to targets in SP than DP conditions. In other words, we found the typical IOR effect, an observation in line with the late inhibition or disengagement hypothesis of Theeuwes et al. [1]. This finding is also in agreement with prior findings of Ansorge et al. [11] with relevant cues. In their study, these authors found a capture effect of the relevant cues when a CTOA of 200 ms was used. This capture effect preceded a subsequent IOR effect. Irrelevant cues only produced reliable IOR effects. Ansorge et al. also observed that IOR started earlier with an irrelevant cue than with a relevant cue. This particular finding could not be observed in the present study. In the present study, among the slowest responses, IOR with irrelevant cues was only numerically but not significantly stronger than IOR with relevant cues. This latter finding is thus also not so well in line with Theeuwes et al.'s disengagement theory, according to which one would have expected stronger disengagement or IOR after irrelevant than after relevant cues. According to disengagement theory, only the stronger disengagement of attention that follows irrelevant cues accounts for seemingly stronger capture effects by relevant than irrelevant cues. Clearly, this prediction of the disengagement theory was not confirmed. In contrast, our results suggested a mixture of early capture differences—with more capture by relevant than irrelevant cues—and a later disengagement effect that was numerically stronger with irrelevant than relevant cues, as two sources contributing to stronger capture effects by relevant than irrelevant cues.

Concerning stronger capture by relevant than irrelevant cues, this was reflected in the N2pc. When we looked at the N2pc as an index of the initial capture of attention, we found a larger overall N2pc. This reflected on average an earlier start of the N2pc elicited by the relevant cue. These findings are in line with prior findings showing an earlier or temporally less variable capture effect and often even a selective capture effect for top-down matching than nonmatching cues [12, 21, 23, 35, 36]. This difference in capture for top-down matching as compared to nonmatching cues is typically assumed to reflect either of two processes: selective top-down tuning to sets of features so that initial capture is restricted to the cues matching the set [12, 37] or less inhibition of attention captured by the top-down matching cue [1, 38]. With the current procedure, we cannot decide which of these interpretations holds true, that is, whether the temporally more variable or trailing onset of the N2pc by the irrelevant cues reflected less initial capture by these cues or a combination of initial capture by the irrelevant cues and proactive inhibition of the irrelevant cues. With respect to the latter, however, we did not find any evidence for strong early proactive inhibition of the irrelevant cues in the form of a Pd. The trailing of the N2pc for irrelevant cues might be a tentative hint for some proactive inhibition. Without any proactive influence, one would expect similar onset times of the N2pc for relevant and irrelevant stimuli (although the initially smaller N2pc for irrelevant stimuli may camouflage its early onset).

In particular, prior studies found proactive inhibition in the form of a Pd when only looking at the fastest responses [2]. In contrast to this finding, early or proactive inhibition was not associated with the fastest responses in the present study. This was evident when we sorted the ERPs as to whether they were recorded in a trial with a quick or slow saccade: among the fast saccades, the N2pcs of irrelevant and relevant cues became even more similar. This means that in the present study, more proactive inhibition would have counteracted the irrelevant cue's N2pc onset in the trials with the slower saccades.

Which factors might account for the differences between the present study and the previous study by McDonald et al. [2]? To reconcile the different findings, results from Kiss and colleagues [27] might be of interest. These authors presented target and distractor simultaneously (similar to [2]) and found proactive inhibition of the irrelevant distractor in the form of a Pd when the display was shown for 200 ms but an N2pc plus subsequent inhibition (again in the form of a Pd but occurring at a later point in time) when the display was presented until a response was given. This might indicate that the irrelevant distractor elicits an N2pc and captures attention when the participants have time for their attention to shift to the target so that the distractor-elicited capture is not masked by a concomitant target-elicited N2pc.

This might also explain why we found an N2pc of the irrelevant cues whereas most contingent-capture studies did not find any evidence for capture by irrelevant singleton distractors (e.g., [21, 39]). With respect to the finding of an N2pc to the irrelevant cue in the present study and its absence in prior studies, a few other procedural differences might also play a role. First of all, the relevant cue was 100% valid (100% SP); that is, it predicted the discrimination target position with certainty. Although there was no discrimination target in the irrelevant target position it is possible that a bit of the general informative value of the relevant cues spilled over to the irrelevant cues. In other words, participants might have inadvertently attended to the irrelevant cue on at least some trials, for example, because they were not paying close enough attention to the color of the first cue. In support of this possibility, it would have been possible to find the relevant cues by the so-called* singleton search strategy* [40, 41]. In fact, the use of two different relevant colors—one (e.g., blue) for the first display's relevant cue and another one (red) for the saccade cue in the second display—might have encouraged our participants to use a singleton search strategy rather than a feature search strategy. A few findings seem to indicate that the use of a top-down set containing two relevant colors leads to the “erroneous” capture of attention by an irrelevant color-singleton distractor in at least some trials (cf. [42–45], but see [46, 47]). In addition, participants might have actively searched for even the irrelevant cues because these cues informed the participants that they would not have to discriminate between the different target orientations and keep the cue's position in mind. The relatively long CTOA might have encouraged this strategy further because it would have allowed sufficient time to first willingly attend to each cue—relevant and irrelevant—and then to return attention to a neutral position after the irrelevant cue and before the onset of the target. Even though this particularity of our procedure might explain why we did find an N2pc for both relevant and irrelevant cues, it is important to note that we were still able to ascertain two things: first, recording EEG we were able to demonstrate capture where behavioral measures only indicated inhibition [11]. Second, we found differences in initial capture for relevant and irrelevant cues. Thus, although one might argue that the difference in the way participants processed relevant and irrelevant cues in our study was only small, our EEG measure was definitely sensitive to it. In sum, we might not have ended the debate over early proactive inhibition for complete prevention of capture once and for all with our study. However, we provide one more piece in the puzzle and another demonstration of the usefulness of combining EEG with behavioral measures to obtain a more complete picture of the processes engaged through a given paradigm.

A further point that needs discussion is the relation between capture and IOR. Originally, IOR was regarded as the reflection of preceding capture [9]. Under this perspective, it would be strange if different degrees of initial capture by relevant versus irrelevant cues ultimately lead to relatively similar degrees of IOR by these stimuli. However, researchers had argued from very early on that capture and certain forms of inhibition could be partly independent processes [48]. Today, it is clear that nonattentional factors like motor inhibition and sensory habituation can also contribute to inhibition [8, 49, 50]. Therefore, it is in principle possible to find similar degrees of late inhibition after different degrees of capture [51–53] or even more inhibition following less capture by an irrelevant stimulus [11]. Along similar lines, Prinzmetal et al. [54] reported that attention capture and IOR are differentially modulated by, on the one hand, the number of potential target locations and, on the other, the presence of distractor stimuli in the target display. Dissociations of attention capture and IOR are also in line with neurophysiological observations suggesting that the two effects arise at different stages of processing and may therefore be modulated differentially (e.g., [55]). In more functional terms, Prinzmetal et al. [54] recently suggested that attention capture may best be described by a serial search mechanism, reminiscent of the attentional spotlight that (at least for top-down matching cues) is first allocated to the cued location and has to be redirected on invalid (DP) trials. IOR, however, may better be accounted for by a decision process in a competitive accumulator model in which the decision to respond to a particular location previously visited by attention is systematically delayed (see also [56]). In conclusion, the two mechanisms proposed for attention capture and IOR are very distinct, supporting the possibility for dissociations.

The present results also show that IOR can be induced by color singletons. Previous studies such as Gibson and Amelio [57] failed to find any evidence for IOR with color singletons, a result that was ascribed to the special role of abrupt onsets for the occurrence of IOR. Here, we show that relevant and even irrelevant color singletons lead to IOR when an eye movement instead of a manual response is used and when the saccadic RT distribution is taken into account. In line with this interpretation, Godijn and Theeuwes [58] and more recently Priess et al. [30] and Ansorge et al. [11] also demonstrated saccadic IOR after color singletons in a saccade task.

## 4. Conclusion

In conclusion, in line with the late inhibition or disengagement theory, we have shown that the irrelevant and the relevant distractor first both captured attention (reflected in their N2pcs) before they were actively inhibited (reflected in saccadic IOR). This lack of proactive inhibition was also found if only the fastest responses were analyzed. However, we found little indication that IOR was stronger after irrelevant than relevant cues. Therefore, it is not likely that disengagement was the only responsible process. Early inhibition (among the slower responses) or contingent capture must have also contributed to the N2pc differences between relevant and irrelevant cues.
